# A nomogram for predicting lymph node metastasis in early gastric signet ring cell carcinoma

**DOI:** 10.1038/s41598-023-40733-1

**Published:** 2023-09-12

**Authors:** Hongwei You, Shengsen Chen, Shi Wang

**Affiliations:** 1 Department of Endoscopy, Zhejiang Cancer Hospital, Hangzhou Institute of Medicine (HIM), Chinese Academy of Sciences, Hangzhou, 310022 Zhejiang China; 2 Postgraduate Training Base Alliance of Wenzhou Medical University (Zhejiang Cancer Hospital), Hangzhou, 310022 Zhejiang China

**Keywords:** Gastric cancer, Gastric cancer, Gastrointestinal models

## Abstract

At present, the risk factors for lymph node metastasis in early gastric signet ring cell carcinoma (SRCC) remain unclear. However, it is worth noting that the LNM rate and prognosis of early gastric SRCC are superior to those of other undifferentiated cancers. With advancements in endoscopic technology, the 5-year survival rate following endoscopic treatment of early gastric cancer is comparable to traditional surgery while offering a better quality of life. The objective of this study was to develop a nomogram that can predict lymph node status in early gastric SRCC before surgery, aiding clinicians in selecting the optimal treatment strategy. A research cohort was established by retrospectively collecting data from 183 patients with early gastric SRCC who underwent radical gastrectomy with lymph node dissection at our hospital between January 2014 and June 2022. The predictors of early gastric signet ring cell carcinoma lymph node metastasis were identified in the study cohort using the least absolute selection and shrinkage operator (Lasso) and multivariate regression analysis, and a nomogram was developed. The discrimination, accuracy, and clinical practicability of the nomogram were assessed using receiver operating characteristic (ROC) curve analysis, calibration curve analysis, and decision curve analysis. The incidence of lymph node metastasis was 21.9% (40/183) overall. Multivariate logistic regression analysis revealed that tumor size and lymphovascular invasion (LVI) were independent risk factors for lymph node metastasis. Lasso regression analysis demonstrated that tumor size, invasion depth, LVI, E-cadherin expression, dMMR, CA242, NLR, and macroscopic type were associated with lymph node metastasis. The integrated discrimination improvement (IDI) (P = 0.034) and net reclassification index (NRI) (P = 0.023) were significantly improved when dMMR was added to model 1. In addition, the area under curve (AUC) (P = 0.010), IDI (P = 0.001) and NRI (P < 0.001) of the model were significantly improved when type_1 was included. Therefore, we finally included tumor size, invasion depth, dMMR, and macroscopic type to establish a nomogram, which had good discrimination (AUC = 0.757, 95% CI 0.687–0.828) and calibration. Decision curve analysis showed that the nomogram had good clinical performance. We have developed a risk prediction model for early gastric signet ring cell carcinoma that accurately predicts lymph node involvement, providing clinicians with a valuable tool to aid in patient counseling and treatment decision-making.

## Introduction

As one of the most common malignant tumors in the world, gastric cancer has seriously threatened human physical and mental health. China has a high incidence of gastric cancer. In 2018, the incidence of gastric cancer in China ranked the third among malignant tumors, while the mortality ranked the second^1^. It is important to note that the prognosis of gastric cancer is closely correlated with the timing of diagnosis and treatment. The 5-year survival rate for early gastric cancer (EGC) exceeds 90%, in contrast to less than 40% for advanced gastric cancer^2^. In recent years, endoscopic resection (ER) techniques such as endoscopic mucosal resection (EMR) and endoscopic submucosal dissection (ESD) have matured significantly. The postoperative 5-year survival rate is comparable to that of traditional surgery, while the quality of life for patients undergoing endoscopic resection is superior. Therefore, endoscopic resection has gradually become the first choice for early gastric cancer^3^. However, the key to the selection of ER is preoperative evaluation of lymph node metastasis. Therefore, the evaluation of lymph node involvement is related to the prognosis and is particularly important for the choice of surgical methods^4^. According to the criteria established by the World Health Organization (WHO) and Japan Classification of Gastric Cancer, gastric cancer can be histologically classified into two types: differentiated carcinoma (DC) and undifferentiated carcinoma (UDC). DC includes papillary adenocarcinoma and moderately to well-differentiated tubular adenocarcinoma, while UDC encompasses mucinous adenocarcinoma, poorly differentiated adenocarcinoma, and signet ring cell carcinoma (SRCC). The degree of differentiation in gastric cancer serves as a crucial reference factor for determining the appropriateness of endoscopic treatment^5–7^. Signet ring cell carcinoma has traditionally been regarded as a highly aggressive tumor with rapid progression and poor prognosis, albeit in cases of advanced gastric SRCC. However, recent advances in our understanding of early and advanced gastric cancer have revealed that early gastric SRCC exhibits lower rates of lymph node metastasis, reduced distant dissemination, and improved prognosis compared to other undifferentiated carcinomas. The latest Japanese guidelines for gastric cancer treatment have expanded the indication of ESD to include undifferentiated intramucosal carcinomas < 2 cm in diameter without ulceration, while the indication of endoscopic resection for early signet ring cell carcinoma (SRCC) remains controversial^8–12^.

Therefore, the aim of this study is to investigate potential correlations between clinicopathological characteristics, gastrointestinal tumor markers, inflammatory markers, biological markers and lymph node metastasis in early gastric SRCC. Additionally, a nomogram will be developed to predict LNM status with the goal of aiding individuals in selecting appropriate treatment strategies for early gastric SRCC.

## Methods

### Patients

The data of patients with T1 gastric SRCC confirmed by postoperative pathology who underwent radical gastrectomy combined with lymph node dissection in Zhejiang Cancer Hospital from January 2014 to June 2022 were retrospectively collected. Exclusion criteria include: (1) multiple gastric cancers; (2) metastatic gastric cancer; (3) gastric stump cancer; (4) patients with preoperative neoadjuvant radiotherapy, chemotherapy or targeted therapy; (5) patients with history of other malignant tumors or incomplete data; (6) patients with diseases affecting peripheral blood cells, such as infection, blood system diseases, etc.

### Definition

Early gastric cancer is defined as the presence of cancer cells limited to the mucosa or submucosa, regardless of lymph node involvement. Based on the Japanese Classification criteria for gastric cancer, we categorized macroscopic types into elevated (type 0–I and 0–IIa), flat (type 0–IIb), and depressed (type 0–IIc and 0–III)^13^. According to anatomical location, the tumors were classified into three groups: upper third (cardia and fundus), middle third (body), and lower third (angle, antrum, and pylorus)^14^. According to the diagnostic criteria established by the World Health Organization (WHO) and American Joint Committee on Cancer (AJCC), pure SRCC is defined as scattered or small clumps of malignant tumor cells containing mucous in their cytoplasm, accounting for more than 50% of the tumor cells. Mixed SRCC refers to tumors composed of partial signet-ring cells. The depth of invasion was classified into two categories: mucosal layer (T1a) and submucosal layer (T1b)^5,15^. Serum tumor markers of digestive tract (CA50, CA724, CA242, Ferritin, CA199, CA125, CEA, AFP) and peripheral blood cell count (white blood cell count, neutrophil count, lymphocyte count, and platelet count) were determined 2 weeks before operation. Then, tumor markers were divided into negative group and positive group according to the critical values of 25 IU/mL, 6.9 U/mL, 20 IU/mL, 22–322 ng/mL, 37 U/mL, 35 U/mL, 5 ng/mL, 8.78 ng/mL. The monocyte lymphocyte ratio (MLR), derived monocyte lymphocyte ratio (dMLR), neutrophil lymphocyte ratio (NLR), derived neutrophil lymphocyte ratio (dNLR) and platelet lymphocyte ratio (PLR) are calculated as follows: MLR = monocyte count to lymphocyte count; dMLR = monocyte count to (white blood cell count − neutrophil count); NLR = neutrophil count to lymphocyte count; dNLR = neutrophil count to (white blood cell count − neutrophil count); PLR = platelet count to lymphocyte count^16–18^. In addition, factors such as tumor size, ulceration, LVI, gender, and age were recorded.

### Immunohistochemistry

Her-2 was located in the cell membrane, and 0 was defined as no staining or < 10% cell membrane staining, 1 + was defined as weak staining with > 10% cell membrane incomplete staining, 2+ was defined as moderate staining with > 10% cell membrane complete staining, 2+ was defined as strong staining with > 10% cell membrane complete staining, and 0 and 1+ were divided into negative group, 2+ and 3+ were divided into positive group^19^. Ki-67 positivity was defined as the presence of tan granules in the nucleus, and the percentage of positive cells was calculated^20^. The E-cadherin (E-cad) staining rate ≤ 50% was defined as the low expression group, and the staining rate > 50% was defined as the high expression group^21^. The absence of any of the four major mismatch repair (MMR) proteins, MLH1, MSH2, MSH6 and PMS2, was defined as positive mismatch repair function deficit (dMMR), and all positive proteins were defined as complete mismatch repair function (pMMR), namely, dMMR negative^22^.

### Ethics statement

This study followed the 1964 Declaration of Helsinki and its subsequent amendments, and was approved by the Ethics Committee of Zhejiang Cancer Hospital (IRB-2023-467). Due to the retrospective nature of this study, the Ethical Review Board of Zhejiang Cancer Hospital waived the required written informed consent for individuals included in the study.

### Statistical analysis

Continuous normal variables were expressed as mean ± standard deviation, while skewed variables were represented by median (interquartile range). The two independent sample t-test and Wilcoxon rank sum tests were employed for comparison. Categorical variables were presented as frequency (%) and analyzed using the Chi-square test or Fisher exact probability method. The candidate variables for multivariate logistic regression analysis were initially screened through univariate analysis^23^. Variable screening was further performed using the least absolute shrinkage and selection operator (LASSO) regression method, with the R and glmnet packages utilized to identify variables with non-zero regression coefficients^24^. The net reclassification index serves as an indicator for comparing the predictive ability of the new model with that of the old model. NRI exhibits higher sensitivity than AUC and places greater emphasis on assessing the relative quality of two diagnostic tests at a specific cut-off value. When attempting to incorporate a new variable into the original model in order to assess whether the predictive capacity of the updated model has improved, it may be challenging to achieve significant enhancements in AUC due to minimal differences between the two models. In this case, NRI can be utilized to more explicitly demonstrate the disparity in predictive efficacy between the new and old models. The Integrated Discrimination Improvement (IDI) is employed to reflect overall model enhancement by considering improvements at different cut points. Compared with AUC, both NRI and IDI offer superior clinical interpretation^25^. If the addition of a variable resulted in a significant improvement in the AUC (or IDI) and NRI of the new model, it was incorporated into the prediction model. NRI and IDI are calculated using R’s PredictABEL package.

**Figure 1:**
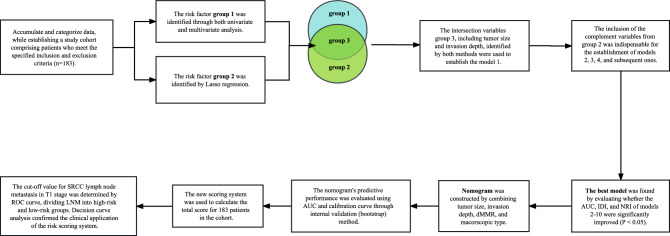
Technology roadmap.

A nomogram was constructed using the rms package in R based on a binary logistic regression model. This multivariate regression model integrates multiple predictor variables and uses line segments with scales to express the relationship between each variable in the prediction model on the same plane according to a certain proportion. The nomogram transforms the intricate regression equation into a visual graph, thereby enhancing the interpretability and convenience of evaluating patients using the prediction model. The accuracy of the nomogram was quantified by calculating the area under the curve (AUC). Nomogram was internally validated using 1000 bootstrap self-sampling^26^. Finally, decision curve analysis (DCA) was performed using the rmda package of R to evaluate the clinical utility of the nomogram^27^. Decision curve analysis is a statistical methodology that enables the assessment of the clinical utility of a prediction model, specifically its potential to enhance patient outcomes by informing decision-making processes. In the univariate analysis, statistical significance was defined as P < 0.1, while in all other analyses it was defined as P < 0.05. The statistical software used for this study included SPSS version 25 and R version 4.2.2, with a technical roadmap presented in Fig. 1.

## Results

### Clinicopathological features of early gastric signet ring cell carcinoma

A total of 183 patients, comprising 94 males and 89 females with a mean age of 54 years (range 22–87), were enrolled from Zhejiang Cancer Hospital. Based on the depth of tumor invasion, mucosal confinement was observed in 98 cases while submucosal invasion was noted in 85 cases. Tumors were distributed in the upper third (6 cases, 3%), middle third (61 cases, 33.3%), and lower third (116 cases, 63.3%) of the sample population. Pure SRCC accounted for 34 cases (18.6%), while mixed SRCC was observed in 149 cases (81.4%). The number of patients with pathologically confirmed LVI was 21. Forty patients (21.9%) exhibited lymph node metastasis as confirmed by postoperative pathology. Among these cases, there were 10 instances (5.5%) of elevated type, 20 instances (10.9%) of flat type, and 153 instances (83.6%) of depressed type (Table 1).

**Table 1:** Relationship between clinicopathological features and lymph node metastasis in early gastric signet-ring cell carcinoma.

Variable	LNM (−) N = 143	LNM (+) N = 40	P
Gender			0.580
Male	75 (52.4)	19 (47.5)	
Female	68 (47.6)	21 (52.5)	
Age (years)	53.95 ± 12.07	56.30 ± 13.89	0.294
Tumor size			**0.002**
≤ 1.45 cm	40 (28)	2 (5)	
> 1.45 cm	103 (72)	38 (95)	
Location			0.667
Upper third	5 (3.5)	1 (2.5)	
Middle third	50 (35)	11 (27.5)	
Lower third	88 (61.5)	28 (70)	
Macroscopic type			**0.014**
Elevated	7 (4.9)	3 (7.5)	
Flat	20 (14)	0 (0)	
Depressed	116 (81.1)	37 (92.5)	
Histological type			**0.042**
Pure	31 (21.7)	3 (7.5)	
Mixed	112 (78.3)	37 (92.5)	
Invasion depth			** < 0.001**
T1a	88 (61.5)	10 (25)	
T1b	55 (38.5)	30 (75)	
Ulcer			0.171
Absent	102 (71.3)	24 (60)	
Present	41 (28.7)	16 (40)	
LVI			** < 0.001**
Absent	137 (95.8)	25 (62.5)	
Present	6 (4.2)	15 (37.5)	
E-cad			**0.061**
Low	10 (7)	7 (17.5)	
High	133 (93)	33 (82.5)	
Her-2			0.104
0/1+	114 (79.7)	27 (67.5)	
2+/3+	29 (20.3)	13 (32.5)	
dMMR			0.587
Negative	138 (96.5)	40 (100)	
Positive	5 (3.5)	0 (0)	
CA50			0.219
Negative	143 (100)	39 (97.5)	
Positive	0 (0)	1 (2.5)	
CA724			0.551
Negative	130 (90.9)	35 (87.5)	
Positive	13 (9.1)	5 (12.5)	
CA242			**0.047**
Negative	143 (100)	38 (95)	
Positive	0 (0)	2 (5)	
Ferritin			1
Negative	127 (88.8)	36 (90)	
Positive	16 (11.2)	4 (10)	
CA199			0.178
Negative	139 (97.2)	37 (92.5)	
Positive	4 (2.8)	3 (7.5)	
CA125			1
Negative	137 (95.8)	39 (97.5)	
Positive	6 (4.2)	1 (2.5)	
CEA			0.208
Negative	141 (98.6)	38 (95)	
Positive	2 (1.4)	2 (5)	
AFP			1
Negative	142 (99.3)	40 (100)	
Positive	1 (0.7)	0 (0)	
Ki-67	0.60 (0.40–0.75)	0.60 (0.43–0.79)	0.881
MLR	0.19 (0.15–0.25)	0.19 (0.14–0.25)	0.902
dMLR	0.15 (0.12–0.19)	0.15 (0.12–0.19)	0.796
NLR	2.36 (1.79–3.09)	2.12 (1.71–2.91)	0.218
dNLR	1.80 (1.47–2.50)	1.64 (1.37–2.29)	0.170
PLR	128.46 (102.17–165.33)	124.46 (103.92–151.61)	0.739

*LVI* lymphovascular invasion, *E-cad* E-cadherin, *Her-2* human epidermalgrowth factor receptor-2, *T1a* mucosal, *T1b* submucosal, *MLR* monocyte count to lymphocyte count, *NLR* neutrophil count to lymphocyte count, *PLR* platelet count to lymphocyte count, *dMLR* monocyte count to (white blood cell count − neutrophil count), *dNLR* neutrophil count to (white blood cell count − neutrophil count).

The bold type indicates statistical significance.

### Univariate and multivariate analysis of clinicopathological features

According to the ROC curve analysis, we determined the optimal cut-off point and categorized tumor size into two groups: ≤ 1.45 cm and > 1.45 cm. Preliminary univariate analysis of the dataset revealed significant associations between lymph node metastasis and several factors including tumor size, depth of invasion, LVI, histological type, macroscopic type, E-cad, and CA242 (Table 1). However, the results of multivariate analysis indicated that only tumor size (OR 4.879, P = 0.047) and LVI (OR 8.324, P < 0.001) exhibited significant associations with lymph node metastasis. Specifically, patients with tumors larger than 1.45 cm and the presence of vascular tumor emboli exhibited a higher propensity for lymph-node metastasis (Table 2). Given the preoperative uncertainty in accurately determining LVI, further investigation was conducted to explore the association between LVI and variables. Notably, the invasion depth (OR 6.441, P = 0.005) exhibited a significant correlation with LVI, thus justifying its inclusion in group 1 (Table 3).

**Table 2:** Multivariate analysis of risk factors for lymph node metastasis in early gastric signet ring cell carcinoma.

Variable	OR	95% CI	P
Tumor size			
≤ 1.45 cm	Reference		
> 1.45 cm	4.879	1.019–23.364	**0.047**
Invasion depth			
T1a	Reference		
T1b	2.273	0.937–5.515	0.069
LVI			
Absent	Reference		
Present	8.324	2.610–26.555	** < 0.001**
Histological type			
Pure	Reference		
Mixed	1.339	0.349–5.141	0.670
Macroscopic type			
Elevated	1.414	0.312–6.414	0.653
Flat	0	0	0.998
Depressed	Reference		
E-cad			
Low	Reference		
High	0.339	0.097–1.186	0.339
CA242			
Negative	Reference		
Positive	2,264,047,285	0	0.999

*LVI* lymphovascular invasion, *E-cad* E-cadherin, *T1a* mucosal, *T1b* submucosal, *OR* odd ratio, *CI* confidence interval.

The bold type indicates statistical significance.

**Table 3:** Multivariate analysis was used to analyze the relationship between LVI and clinicopathological factors.

Variable	OR	95% CI	P
Tumor size			
≤ 1.45 cm	Reference		
> 1.45 cm	1.628	0.331–7.996	0.549
Invasion depth			
T1a	Reference		
T1b	6.441	1.769–23.446	**0.005**
Histological type			
Pure	Reference		
Mixed	1.339	0.349–5.141	0.670
Macroscopic type			
Elevated	0.465	0.053–4.088	0.490
Flat	0	0	0.998
Depressed	Reference		
E-cad			
Low	Reference		
High	0.775	0.183–3.288	0.729
CA242			
Negative	Reference		
Positive	2.871	0.167–49.283	0.467

*LVI* lymphovascular invasion, *E-cad* E-cadherin, *T1a* mucosal, *T1b* submucosal, *OR* odd ratio, *CI* confidence interval.

The bold type indicates statistical significance.

### LASSO regression analysis

**Figure 2:**
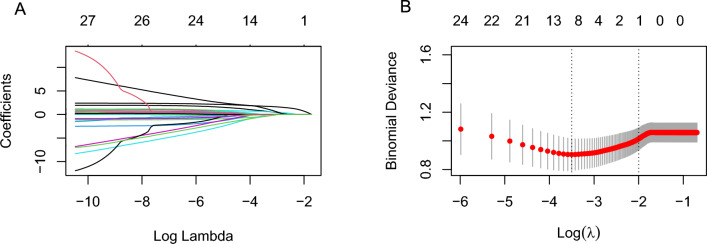
Using Lasso regression to screen variables. (**A**) The path diagram of regression coefficients of 28 variables. With the increase of λ value, the punishment intensity increases continuously, and the regression coefficients of 28 variables in the model will be gradually compressed to 0. (**B**) 10-fold cross-validation curve, the left dashed line is the λ value corresponding to the minimum mean square error, and the right dashed line is the λ value corresponding to the minimum mean square error-1 standard error.

**Figure 3:**
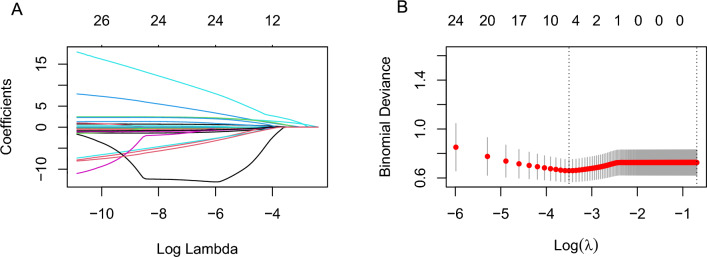
LASSO regression was used to evaluate the relationship between LVI and clinical parameters. (**A**) The path diagram of regression coefficients of 27 variables. (**B**) 10-fold cross-validation curve.

In the dataset, the depressed type was designated as the reference group, while the macroscopic type was recoded into two binary variables: elevated and flat types. The control group was defined as the lower 1/3 of the tumor location and subsequently transformed into two dummy variables: upper 1/3 and middle 1/3. As the penalty coefficient λ increased, the regression coefficient of the independent variable gradually diminished until it eventually reached zero. The independent variables were verified using tenfold cross-validation. When λ = 0.030 and log (λ) =  − 3.502, we identified eight non-zero coefficient variables associated with lymph node metastasis, with the following regression coefficients: tumor size (0.741), invasion depth (0.615), LVI (1.755), E-cad (− 0.423), dMMR (− 0.241), CA242 (1.362), NLR (− 0.069), and type_1 (− 0.454) (Fig. 2). The association between LVI and each parameter was assessed, resulting in the identification of five variables significantly associated with LVI. The corresponding regression coefficients were as follows: histological type (0.490), invasion depth (1.180), Her-2 status (0.051), Ki-67 expression level (0.514), and CA50 level (1.988) (Fig. 3). Consequently, histological type, Her-2, Ki-67, and CA50 were included in group 2.

### The nomogram was established and the best model was fou*nd*

The base model (Model 1) was constructed by incorporating two variables, namely tumor size and invasion depth, which are closely associated with lymph node metastasis. Subsequently, model 2 was constructed by incorporating E-cad based on model 1. Model 3 was developed by including DMMR, while CA242 was integrated to construct model 4. Model 5 encompassed NLR, and Type_1 was incorporated to establish model 6. Furthermore, histological type contributed to the construction of model 7, followed by Her-2 in model 8. Ki-67 played a role in constructing model 9, and finally, CA50 was included in the development of model 10. The AUC, IDI, and NRI of the other models were compared to model 1 as the reference. The results indicated that model 3 exhibited a significant improvement in both IDI (P = 0.034) and NRI (P = 0.023) when compared to model 1. Additionally, model 6 demonstrated a significant enhancement in AUC (P = 0.010), IDI (P = 0.001), and NRI (P < 0.001) relative to model 1. Furthermore, the NRI values of models 4, 7, and 10 as well as the IDI value of model 5 exhibited improvement. Thus, we can conclude that models 3 and 6 possess superior predictive power compared to Model 1. Additionally, both dMMR and macroscopic type can be regarded as robust predictors for lymph node metastasis in conjunction with tumor size and invasion depth (Table 4).

**Table 4:** Comparison of different models for predicting the risk of lymph node metastasis.

	AUC (95% CI)	P value	IDI% (95% CI)	P value	NRI% (95% CI)	P value
Model 1	0.73 (0.66–0.81)	Reference		Reference		Reference
Model 2	0.75 (0.68–0.83)	0.123	0.02 (− 0.01 to 0.05)	0.133	0.21 (− 0.04 to 0.46)	0.099
Model 3	0.74 (0.67–0.81)	0.114	0.01 (0 to 0.01)	**0.034**	0.07 (0.01 to 0.13)	**0.023**
Model 4	0.74 (0.66–0.82)	0.149	0.02 (− 0.02 to 0.07)	0.262	− 0.26 (− 0.47 to − 0.05)	**0.017**
Model 5	0.76 (0.68–0.85)	0.118	0.03 (0.01 to 0.05)	**0.005**	0.32 (− 0.02 to 0.65)	0.064
Model 6	0.75 (0.68–0.82)	**0.010**	0.02 (0.01 to 0.03)	**0.001**	0.28 (0.17 to 0.39)	** < 0.001**
Model 7	0.74 (0.67–0.82)	0.158	0.01 (− 0.01 to 0.02)	0.524	0.28 (0.07 to 0.50)	**0.009**
Model 8	0.75 (0.67–0.83)	0.260	0.01 (− 0.01 to 0.03)	0.289	0.24 (− 0.07 to 0.56)	0.133
Model 9	0.74 (0.66–0.82)	0.742	0 (− 0.01 to 0)	0.854	0.10 (− 0.25 to 0.46)	0.557
Model 10	0.74 (0.66–0.81)	0.319	0.01 (− 0.02 to 0.04)	0.435	− 0.31 (− 0.49 to − 0.12)	**0.002**

*AUC* area under curve, *IDI* integrated discrimination improvement, *NRI* net reclassification index, *CI* confidence interval.

Model 1 = Tumor size + Invasion depth.

Model 2 = Model 1 + E-cad.

Model 3 = Model 1 + dMMR.

Model 4 = Model 1 + CA242.

Model 5 = Model 1 + NLR.

Model 6 = Model 1 + type_1.

Model 7 = Model 1 + histological type.

Model 8 = Model 1 + Her-2.

Model 9 = Model 1 + Ki-67.

Model 10 = Model 1 + CA50.

The bold type indicates statistical significance.

### Nomogram for predicting lymph node metastasis

**Figure 4:**
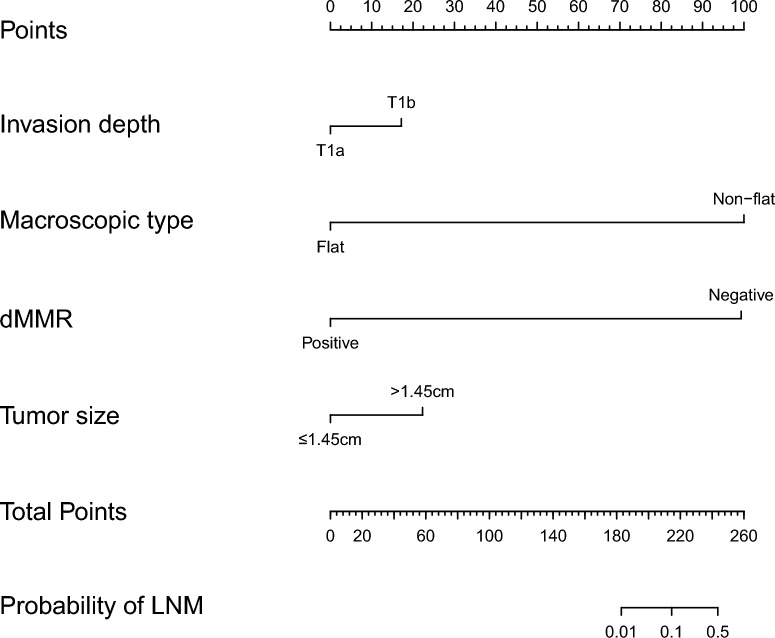
A nomogram was developed to predict lymph node metastasis in early gastric signet ring cell carcinoma, wherein the contribution of each variable to lymph node metastasis was represented by a vertical line corresponding to its value level and assigned a score. Subsequently, the total score was calculated and marked on the total score line, followed by drawing a vertical line intersecting with the prediction probability line to determine the probability of lymph node metastasis. *LVI* lymphovascular invasion, *T1a* mucosal, *T1b* submucosal.

**Figure 5:**
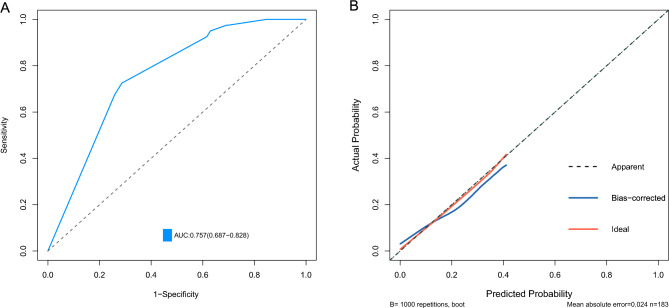
Internal validation of Nomogram. (**A**) Receiver operating characteristic (ROC) curves of the study cohort. (**B**) Calibration curve after 1000 Bootstrap self-sampling in the study cohort.

A nomogram was constructed by incorporating four variables, namely tumor size, invasion depth, dMMR status, and macroscopic type. Each risk factor was assigned a score for every patient based on its contribution to the outcome event (Fig. 4). The nomogram was validated using the internal validation method (bootstrap), and the model demonstrated an area under the receiver operating characteristic curve (AUC) of 0.757 (95% CI 0.687–0.828) (Fig. 5A). The calibration curve of this nomogram exhibited excellent concordance with the model (Fig. 5B).

### Clinical application of nomogram for the risk of LNM metastasis

**Figure 6:**
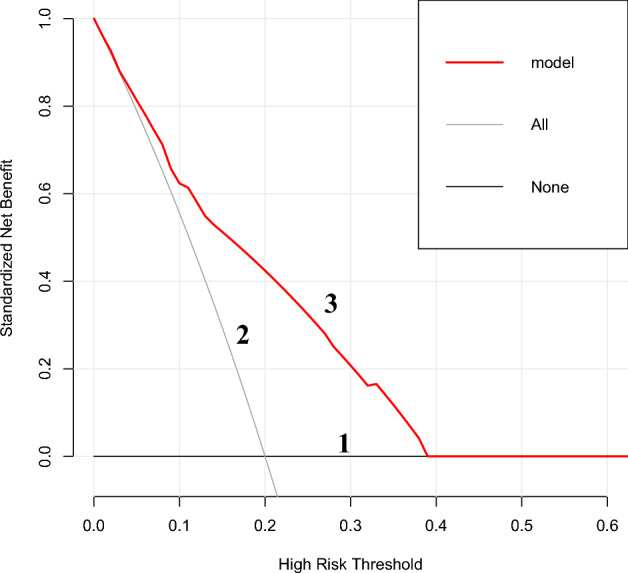
Decision Curve of Nomogram. The benefit line in *Line 1* corresponds to the point of 0.0 on the vertical axis, indicating that no treatment for all patients results in a net clinical benefit of 0; The net clinical benefit curve for all patients is shown in *Line 2*; The net benefit curve for treating patients based on the clinical prediction model is shown in *Line 3*.

The scores for each predictor variable (invasion depth, tumor size, dMMR, macroscopic type) were derived from the nomogram and subsequently aggregated to compute individual patient's total score. The total score ranged from 0 to 238. By employing the ROC curve analysis to identify the maximum Youden index, an optimal cut-off point of 229.5 was determined, yielding a corresponding sensitivity of 0.725 and specificity of 0.713. According to the optimal cut-off point, patients with a total score ≤ 229.5 were classified as having a low risk of lymph node metastasis, while those with a total score > 229.5 were classified as having a high risk of lymph node metastasis. Furthermore, we conducted a decision curve analysis for our model, which demonstrated favorable net benefit within the threshold probability range of 0% to 38%, indicating strong clinical utility (Fig. 6).

## Discussion

The signet ring cell carcinoma is a distinct variant of gastric cancer characterized by an abundant presence of mucin. It represents a highly aggressive malignancy, displaying remarkable invasiveness and rapid disease progression. The majority of patients remain asymptomatic until the disease reaches an advanced stage. However, with the widespread adoption of endoscopic screening, early detection of gastric cancer has become increasingly achievable, and surgical intervention remains the primary treatment modality. Numerous recent studies have consistently demonstrated that the incidence of lymph node metastasis in early gastric SRCC is comparable to that observed in differentiated gastric cancer. Consequently, endoscopic resection emerges as a safe and feasible approach for managing early SRCC^9,11,28^. Revised absolute indications for ESD resection of undifferentiated carcinoma in the sixth edition of the Japanese Gastric Cancer Treatment Guidelines are as follows: intramucosal undifferentiated carcinoma with a diameter ≤ 2 cm and without ulcerative manifestations^12, 29^. However, there is currently no authoritative guideline available for the implementation of endoscopic resection in early gastric SRCC. Therefore, the objective of this study was to identify the risk factors associated with lymph node metastasis in early gastric signet ring cell carcinoma and develop a nomogram that can assist clinicians in selecting an appropriate treatment strategy.

Hu et al.^30^ conducted both univariate and multivariate analyses on a cohort of 160 patients with early-stage SRCC, revealing that a tumor diameter greater than 2 cm was significantly associated with an increased risk of lymph node metastasis. Japanese gastric cancer treatment guidelines specify the indications for ESD of UDC, which require a maximum diameter of ≤ 2 cm. However, Pyo et al.^31^ further stratified the patients into two subgroups based on a tumor size threshold of 1.7 cm. The findings revealed that an elevated tumor type with a size ≥ 1.7 cm and LVI positivity were independent predictors for lymph node metastasis. Conversely, in our study, we observed that tumors larger than 1.45 cm were associated with an increased risk of lymph node metastasis. Hence, does this imply that early-stage SRCC exclusively restricts the penetration of epithelial layer by smaller tumors, preventing invasion into blood vessels and lymphatic vessels within the lamina propria and subsequent lymph node metastasis? Conversely, infiltration of tumors into the submucosal layer is more prone to induce lymph node metastasis due to its abundant blood and lymphatic vessels; as tumor invasion deepens, the likelihood of lymph node metastasis increases. Previous studies have demonstrated that ulceration is an independent risk factor for lymph node metastasis in early undifferentiated gastric cancer, and guidelines stipulate that ESD treatment should not be administered to patients with undifferentiated cancer who present with ulceration^32,33^. Interestingly, Tong et al.^9^ reviewed the pathological features of 422 cases of early gastric cancer and found that ulceration was not associated with lymph node metastasis of early gastric SRCC. It was hypothesized that early signet ring cell carcinoma shared similar pathological features with differentiated gastric cancer, and thus, treatment strategies may overlap. The guidelines for differentiated intramucosal carcinoma with ulceration recommend a tumor size of ≤ 3 cm, which aligns with the findings of our study. LVI denotes the presence of malignant cells within lymphatic or blood vessels, representing a crucial step in the invasion-metastasis cascade. When morphological identification is conducted adjacent to cancer, LVI serves as a robust predictor of metastatic potential^34^. Previous studies have demonstrated that LVI plays a significant role in promoting lymph node metastasis in early gastric signet ring cell carcinoma^11, 33, 35^. And this was also confirmed by our study. The incidence of LVI was 4.2% in the LNM-negative group and 37.5% in the LNM-positive group.

**Figure 7:**
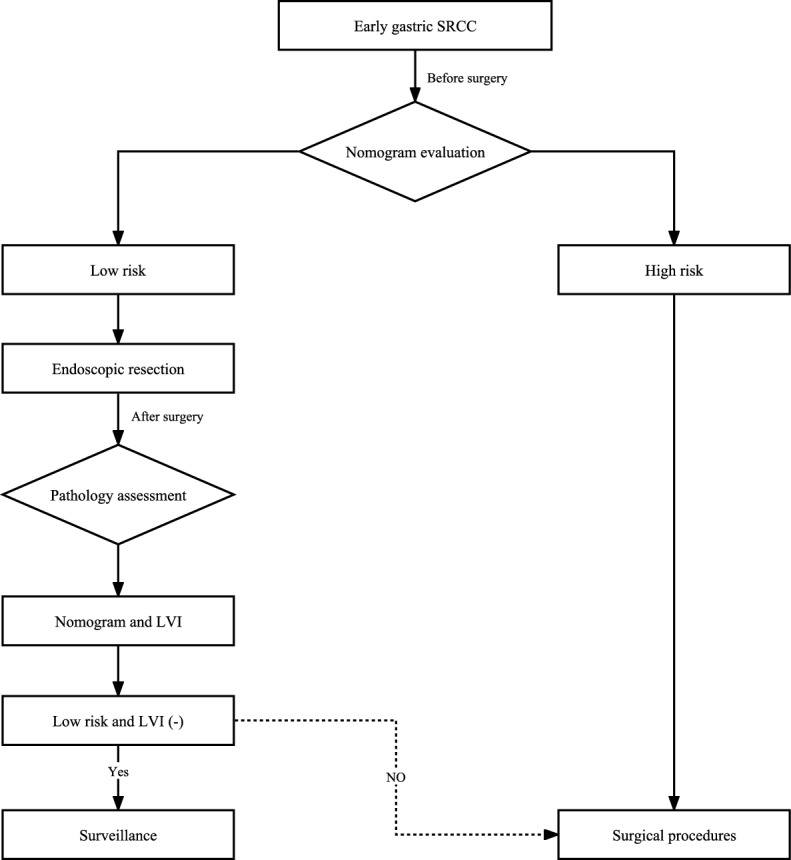
Treatment selection for early gastric signet ring cell carcinoma patients.

In conjunction with previous findings and our results, tumor size, invasion depth, macroscopic type, and dMMR are potential predictive factors for lymph node metastasis in early gastric signet ring cell carcinoma. Consequently, we have developed a nomogram that incorporates tumor size, invasion depth, macroscopic type, and dMMR to accurately forecast lymph node metastasis in early gastric SRCC. By establishing a cutoff value of 229.5, patients can be effectively classified into low-risk or high-risk groups. The overall rate of lymph node metastasis was 21.9% (40/183). Nomogram analysis identified 70 patients in the high-risk group, of whom 29 (41.4%) had lymph node metastasis, while only 11 (9.7%) of the 113 patients in the low-risk group had lymph node involvement. However, with the inclusion of LVI, the rate of lymph node metastasis in the high-risk group increased to 42.7% (32/75), whereas it decreased to 7% in the low-risk group. This predictive model provides clinicians with valuable guidance for treatment decisions. The patients with early SRCC evaluated by preoperative nomogram as low-risk patients underwent endoscopic resection, and the patients with high-risk SRCC (tumor > 1.45 cm, submucosa invasion, non-flat type and dMMR negative) underwent surgical resection. Patients classified as low risk preoperatively were subjected to postoperative evaluation using a nomogram and double assessment for LVI based on the pathological report. Follow-up was conducted for patients with low risk and absence of LVI, while additional surgical intervention was performed in cases where these criteria were not met (Fig. 7). Currently, there is a dearth of precise criteria for early SRCC to identify the risk factors associated with LNM. Therefore, this predictive model could prove beneficial in screening patients who are suitable for endoscopic submucosal dissection treatment and avoiding unnecessary surgery.

As most of the pathological features cited in both literature and our study were obtained post-surgery, there is a potential for preoperative evaluation bias. Furthermore, discrepancies between preoperative biopsy and postoperative pathology diagnosis are not uncommon. Therefore, we employed a method of combined preoperative and double postoperative evaluations to minimize such deviations. However, there are certain limitations associated with this study. Firstly, it should be noted that this is a retrospective study conducted solely at a single institution. Moreover, due to the limited sample size, external validation has not been performed, which may introduce selection bias. In future investigations, it would be advantageous to incorporate data from multiple centers for external validation of the model. Additionally, there is a lack of long-term survival follow-up data regarding early gastric signet ring cell carcinoma after endoscopic treatment. Therefore, future studies with a large sample size and long-term follow-up data are necessary to assess the rate of lymph node metastasis in prospective patients.

## Data Availability

The datasets used and/or analysed during the current study available from the corresponding author on reasonable request.

## Abbreviations

SRCC: Signet ring cell carcinoma
LNM: Lymph node metastasis
ROC: Receiver operating characteristic
NRI: Net reclassification index
IDI: Integrated discrimination improvement
EGC: Early gastric cancer
ER: Endoscopic resection
EMR: Endoscopic mucosal resection
ESD: Endoscopic submucosal dissection
OR: Odd ratio
CI: Confidence interval
LASSO: Least absolute selection and shrinkage operator
DCA: Decision curve analysis
